# Prolonged enoxaparin therapy compared with standard-of-care antithrombotic therapy in opiate-treated patients undergoing primary percutaneous coronary intervention

**DOI:** 10.1080/09537104.2020.1779925

**Published:** 2020-06-16

**Authors:** Wael Sumaya, William A.E. Parker, Heather M. Judge, Ian R. Hall, Rachel C. Orme, Zulfiquar Adam, James D. Richardson, Alexander M.K. Rothman, Kenneth P. Morgan, Julian P. Gunn, Robert F. Storey

**Affiliations:** 1 Department of Infection, Immunity and Cardiovascular Disease, Medical School, University of Sheffield, Sheffield, UK; 2 South Yorkshire Cardiothoracic Centre, Northern General Hospital, Sheffield Teaching Hospitals NHS Foundation Trust, Sheffield, UK

**Keywords:** Enoxaparin, glycoprotein IIb/IIIa inhibitors, P2Y_12_ inhibitor, primary percutaneous coronary intervention, ST-ELEVATION myocardial infarction, unfractionated heparin

## Abstract

A novel enoxaparin regimen consisting of intra-arterial bolus (0.75 mg/kg) followed by intravenous infusion (0.75 mg/kg/6 hours) has been developed as a possible solution to the delayed absorption of oral P2Y_12_ inhibitors in opiate-treated ST-elevation myocardial infarction (STEMI) patients undergoing primary angioplasty. We aimed to study the feasibility of this regimen as an alternative to standard-of-care treatment (SOC) with unfractionated heparin ± glycoprotein IIb/IIIa antagonist (GPI). One hundred opiate-treated patients presenting with STEMI and accepted for primary angioplasty were randomized (1:1) to either enoxaparin or SOC. Fifty patients were allocated enoxaparin (median age 61, 40% females) and 49 allocated SOC (median age 62, 22% females). One developed stroke before angiography and was withdrawn. One SOC patient had a gastrointestinal bleed resulting in 1 g drop in hemoglobin and early cessation of GPI infusion. Two enoxaparin patients had transient minor bleeding: one transient gingival bleed and one episode of coffee ground vomit with no hemoglobin drop or hemodynamic instability. Two SOC and no enoxaparin group patients had acute stent thrombosis. These preliminary data support further study of this novel 6-hour enoxaparin regimen in opiate-treated PPCI patients.

## Introduction

Dual antiplatelet therapy is essential to safely perform percutaneous coronary intervention (PCI) [1]. Potent P2Y_12_ inhibitors (ticagrelor/prasugrel) are preferred to clopidogrel in acute coronary syndrome (ACS) patients [1–3] in view of their more rapid and consistent antiplatelet effect [4–6]. However, in opiate-treated patients undergoing primary PCI (PPCI) for ST-elevation myocardial infarction (STEMI), absorption and, therefore, onset of action can be delayed by up to 6–8 hours [7–9]. This can increase the risk of acute thrombotic complications and so strategies to deal with this issue are needed [10,11]. The negative interaction between opiates and oral P2Y_12_ inhibitors was also noted in patients with non-ST-elevation ACS undergoing early catheterization and this may have resulted in increased thrombotic risk [12]. Available solutions have been limited by added cost and increased risk of bleeding [13,14]. For example, we have recently demonstrated a reduction in acute stent thrombosis with an institutional protocol employing routine use of a 6-hour regimen of tirofiban in opiate-treated patients undergoing PPCI but at the expense of more bleeding [10,11]. Enoxaparin, in conjunction with antithrombin, directly inhibits thrombin-induced platelet activation and also inhibits thrombin generation through inhibition of factor Xa, thus indirectly reducing thrombin-induced platelet activation *in vivo* [15]. A novel regimen consisting of a bolus intra-arterial (IA) enoxaparin (0.75 mg/kg) followed by an intravenous (IV) enoxaparin infusion (0.75 mg/kg/6 hours) has been shown to result in sustained antithrombotic effects 6 hours post-PPCI [15]. We hypothesize that this enoxaparin-based regimen is sufficient to circumvent the risk associated with delayed absorption of oral antiplatelet therapy. A bolus dose of enoxaparin (0.5 to 0.75 mg/kg) is acceptable as an alternative to a bolus of unfractionated heparin (UFH) in patients undergoing PPCI [3,16]. Similarly, the safety and efficacy of subcutaneous enoxaparin (1 mg/kg twice daily) are well established in patients with non-ST-elevation ACS [17]. An infusion of enoxaparin following the bolus dose in the context of PPCI is more desirable than a subcutaneous approach, given the more rapid onset of action and avoidance of excessive peak anti-Xa levels [18]. In this study, we aimed to assess the feasibility of using this enoxaparin regimen (IV/IA bolus enoxaparin 0.75 mg/kg + IV infusion 0.75 mg/kg/6 hours) as an alternative to the local SOC, which consists of IV/IA UFH bolus ± the glycoprotein IIb/IIIa inhibitor (GPI) tirofiban in opiate-treated patients undergoing PPCI.

## Materials and Methods

### Study Design and Ethical Considerations

This was a single-center, open-label, feasibility, randomized controlled trial. All study patients provided informed consent according to a protocol approved by the local research ethics committee (18/YH/0108) and the Medicines and Healthcare Products Regulatory Agency (MHRA), UK. The trial was registered at http://clinicaltrials.gov (unique identifier NCT03568838).

### Study Population, Randomization, and Intervention

Patients presenting with STEMI, pre-treated with opiates and accepted for PPCI were recruited if they met the inclusion and exclusion criteria as detailed in the supplement. Patients were randomized, after witnessed informed verbal consent, to either SOC or enoxaparin. Randomization was undertaken using sealed envelopes. Envelopes were opaque, tamper-proof, and prepared independently using random numbers from the Documenta Geigy scientific tables. Treatment was administered after insertion of the IA (intra-arterial) sheath if the diagnosis was confirmed. Patients allocated enoxaparin received IA bolus of enoxaparin (0.75 mg/kg) followed by an IV enoxaparin infusion (0.75 mg/kg/6 h), as previously described [15]. We planned to stop the infusion at 3 hours in patients with significant renal impairment (eGFR<30 ml/min/1.73 m^2^). GPI use was prohibited unless for bailout (no-reflow).

Patients allocated SOC received treatment at the discretion of the treating cardiologist. This consisted of weight-adjusted bolus dose of UFH 50–70 IU/kg + a 6-hour regimen of tirofiban (or UFH 70 IU/kg alone if concerns about bleeding risk). This regimen of tirofiban consisted of tirofiban 25 mcg/kg over 3 minutes (or 6 minutes if weight > 120 kg) followed by maintenance dose of 0.15 mcg/kg/min for patients with eGFR ≥30 ml/min/1.73 m^2^ for 6 hours and half these doses if eGFR<30 ml/min/1.73 m^2^.

As soon as possible following PPCI, written informed consent was obtained. If a patient died before obtaining written consent, data obtained under verbal consent were retained, as approved by the research ethics committee.

Patients were followed up at 24 hours and 30 days. The primary feasibility outcome was recruitment rate. The primary clinical outcome was bleeding events of at least type 2 severity as classified by the Bleeding Academic Research Consortium (BARC) [19]. Secondary endpoints included death, definite or probable stent thrombosis as defined by the Academic Research Consortium [20], and ST-segment resolution post-PPCI. Clinical events were adjudicated by two investigators independently (WS and RFS). ST-segment resolution was calculated within 1 hour of PPCI by an investigator (WAEP) blinded to treatment allocation, using standard criteria [21,22].

### Statistical Analysis

Continuous data are presented as median (quartile 1, quartile 3) and compared using the Mann–Whitney test. Categorical data are presented as number (%) and compared using the Chi-square test. Results with *P*-values < 0.05 were considered statistically significant. Statistical analyses were performed using GraphPad Prism 7 for Mac OS X. This was a feasibility study and clinical data are only reported as pilot data since the study was not powered for clinical outcomes.

## Results

### Baseline, Procedural Characteristics, and Feasibility Outcomes

**Figure 1.**
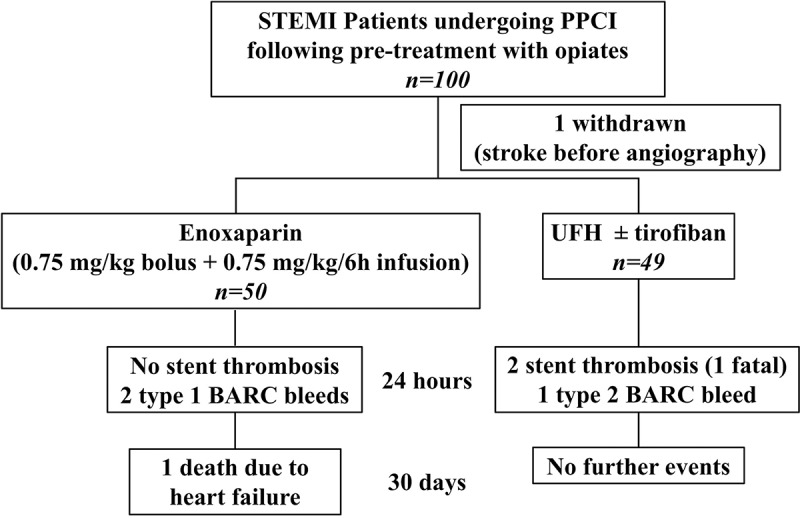
Study flowchart STEMI: ST-elevation myocardial infarction; PPCI: primary percutaneous coronary intervention; UFH: unfractionated heparin

One hundred and eighty-nine patients were screened between end of June 2018 and beginning of March 2019. Thirty-three patients did not meet the eligibility criteria, two declined to participate, and the dedicated research team were not available to obtain verbal consent in 54 patients. Patients meeting eligibility criteria and able to provide consent (n = 100) were recruited: 50 in the enoxaparin group, 49 in the SOC group, and one was withdrawn as he developed stroke after verbal consent but before coronary angiography or receiving any treatment (Figure 1). All patients had received morphine and/or diamorphine (Table I). Baseline demographics, risk factors, and procedural characteristics were well matched between the two treatment groups (Table I). There were slightly more females in the enoxaparin group compared to the SOC group (*P* = .06). All patients received oral aspirin and either ticagrelor (98 patients) or prasugrel (1 patient). Radial artery access was used in the majority of patients (Table I). Three enoxaparin patients were switched to tirofiban for bailout (poor or no re-flow). Forty of the 49 SOC patients received tirofiban, three of whom had poor or no re-flow.

**Table I.** Baseline and procedural characteristics

	Enoxaparin (n = 50)	Standard-of-Care (n = 49)	P value
**Demographics**			
Age (yrs)	61 (55–73)	62 (35–71)	0.52
Female sex	20 (40%)	11 (22%)	0.06
BMI (kg/m^2^)	28 (25–30)	28 (26–30)	0.54
Race			0.22
White	49 (98%)	47 (96%)	
Black	1 (2%)	0 (0%)	
Asian	0 (0%)	2 (4%)	
**Background history**			
Current or past smoker	33 (66%)	36 (73.5%)	0.42
Hypertension	25 (50%)	21 (43%)	0.48
Dyslipidaemia	20 (40%)	19 (39%)	0.9
Diabetes mellitus	12 (24%)	10 (20%)	0.67
Previous MI	8 (16%)	5 (10%)	0.39
Previous PCI	7 (14%)	3 (6%)	0.19
Previous CABG	0 (0%)	1 (2%)	0.31
Cardiac failure	1 (2%)	1 (2%)	0.98
Cerebrovascular disease	2 (4%)	1 (2%)	0.57
Peripheral arterial disease	1 (2%)	0 (0%)	0.32
**Procedural characteristics**			
Anterior territory	22 (45%)	17 (35%)	0.3
Radial access	44 (88%)	44 (90%)	0.78
Ticagrelor 180 mg	50 (100%)	48 (98%)	0.3
Prasugrel 60 mg	0 (0%)	1 (2%)	
GPI use (tirofiban)	3 (6%)	40 (82%)	<0.0001
Slow or no re-flow	3 (6%)	3 (6%)	0.97
Morphine (mg)	6.5 (4.75–10)	7.5 (5–10)	0.41
Diamorphine (mg)	5 (5–7.5)	3.75 (2.5–5)	0.08
Antiemetic treatment	42 (84%)	40 (82%)	0.75
Pain to balloon (mins)	176 (148–250)	210 (157–330)	0.23
Call to balloon (mins)	128 (110–161)	126 (105–182)	0.75
Door to balloon (mins)	44 (32–58)	42 (30–57)	0.72
Number of stents	1 (1–2)	1 (1–2)	0.87
Stent diameter (mm)	3 (3–3.5)	3.5 (3–4)	0.16
Stent length (mm)	23 (15–26)	23 (18–30)	0.17

Data presented as median (interquartile range) for continuous variables and number (%) for categorical variables. BMI: body mass index; MI: myocardial infarction; PCI: percutaneous coronary intervention; CABG: coronary artery bypass graft surgery; GPI: glycoprotein IIb/IIIa inhibitor. *P* values were calculated using Chi-square or Mann–Whitney test as appropriate.

### Clinical Outcomes and ST-segment Resolution

*Bleeding events*: One out of 49 SOC patients had a type 2 BARC bleeding event. The patient vomited fresh blood resulting in 1 g/dL fall in hemoglobin. The tirofiban infusion was stopped.

Two out of 50 enoxaparin patients had type 1 BARC bleeding events. One had a transient gingival bleed and another had an episode of coffee ground vomit. These did not result in a fall in hemoglobin, hemodynamic instability, or change of treatment.

*Acute stent thrombosis*: Two out of 49 SOC patients developed acute stent thrombosis. One had successful PPCI to the right coronary artery (RCA) and was treated with UFH without GPI; approximately 40 minutes after UFH, the patient developed recurrent chest pain and ST-elevation, diagnosed as acute stent thrombosis, and died following decision to manage this conservatively due to age and co-morbidities that had not been appreciated at the time of emergency angiography. The other patient had PPCI to an ectatic RCA but had TIMI-2 flow with high thrombus burden that was treated with UFH and a 48-hour tirofiban infusion. About 8 hours following PPCI, this patient developed further chest pain with recurrent ST-elevation, and repeat angiography confirmed stent thrombosis. However, this was not treated as, by the time repeat angiography was performed, the patient was pain-free and had already developed collateral supply. No patient in the enoxaparin group suffered an acute thrombotic event.

*30-day outcomes*: Except for the 2 SOC patients with acute stent thrombosis who had evidence of recurrent STEMI, no patients suffered recurrent ACS within 30 days and no patient suffered a stroke. In the SOC group, there were no further deaths in addition to the one patient who died as a result of acute stent thrombosis. In the enoxaparin group, one patient was re-admitted with heart failure approximately 2 weeks after discharge and subsequently died from left ventricular failure.

Clinical outcomes are summarized in Figure 1.

**Figure 2.**
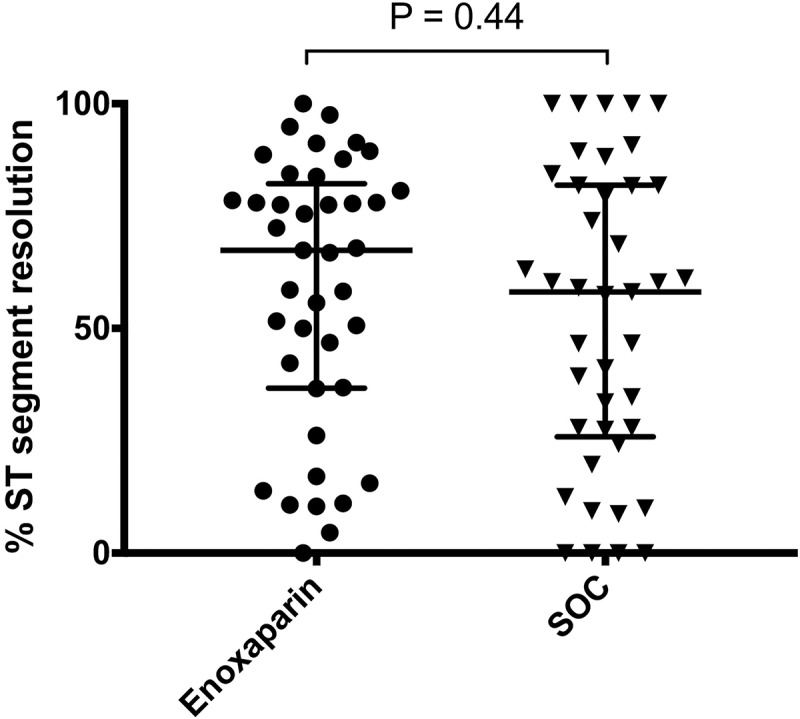
Percentage ST-segment resolution within 1 hour following primary percutaneous coronary intervention lines represent median and interquartile range. SOC: standard-of-care

There was no significant difference in % of ST segment resolution between enoxaparin and SOC groups (*P* = .44) (Figure 2).

## Discussion

In previous work, we demonstrated that an enoxaparin regimen consisting of 0.75 mg/kg bolus followed by intravenous infusion of 0.75 mg/kg over 6 hours resulted in sustained antithrombotic effects throughout the infusion, up to 6 hours post PPCI [15]. This is the first randomized feasibility trial to suggest feasibility of this novel regimen in opiate-treated STEMI patients undergoing PPCI, in comparison with standard parenteral antithrombotic therapy, and provides reassuring pilot data to support future trials.

Bolus enoxaparin (0.5–0.75 mg/kg) is approved as an alternative to bolus UFH to support the PPCI procedure [3,16]. IV enoxaparin half-life is 1–2 hours [23]. The novel approach to follow the bolus dose with an infusion has been designed to bridge the gap in antithrombotic therapy shortly after PPCI. This may represent an inexpensive solution to the delayed antiplatelet effect of oral therapy. As an anticoagulant, enoxaparin inhibits thrombin generation and, in conjunction with plasma antithrombin, thrombin-induced platelet activation [15].

It should be emphasized that clinical outcomes described here are only hypothesis-generating, as our study lacks sufficient power to demonstrate efficacy or safety. Furthermore, the rate of stent thrombosis in the SOC patients (4%) was higher than expected and likely due to chance since we have recently shown that introduction of our institutional guideline for the use of a 6-hour tirofiban regimen in opiate-treated patients undergoing PPCI was associated with a reduction in 30-day stent thrombosis rates to 0.6% [11]. These results, however, highlight the potential caveats with current SOC. Although the risk of stent thrombosis is small, consequences, as was the case in one of our patients, can be catastrophic. Furthermore, one patient developed stent thrombosis despite treatment with GPI, indicating that GPI may not be successful at preventing stent thrombosis in poor flow conditions. We also observed one patient developed fresh hematemesis while receiving GPI. This highlights the potential risk of using routine GPI in combination with heparin and dual oral antiplatelet therapy in view of the expected severe effect on hemostasis of this combination.

The open-label design of the study is another limitation. However, due to the nature of the study recruiting STEMI patients, it was logistically challenging to blind investigators/patients from treatment allocation and no pharmaceutical company support was available to support a double-blind approach.

The lack of difference in ST resolution between the two strategies suggests a lack of differential effect on impairment of the microvascular circulation. Other work has shown that potent P2Y_12_ inhibition at the time of PPCI with cangrelor did not translate into improvement in infarct size or microvascular circulation [24]. As such, it appears that antithrombotic treatment is needed as a preventative therapy rather than as an intervention to aid microvascular circulation. Although cangrelor may be an attractive option to circumvent the delayed absorption of ticagrelor or prasugrel, it has only been studied as a 2–4 hour infusion, which may not be sufficient to cover the delayed onset of action of oral P2Y_12_ inhibitors in all patients [14,24]. Furthermore, it may not be affordable in many healthcare settings to offer cangrelor as a routine 6-hour infusion to opiate-treated STEMI patients undergoing PPCI. Selatogrel is a subcutaneous P2Y_12_ inhibitor with rapid onset of action that is in phase II development and may provide an alternative treatment in the future [25].

Strategies to expedite absorption of oral therapy have been explored. These include using higher loading doses, chewing tablets, or using prokinetic antiemetic drugs [26–29]. However, these have only resulted in marginal acceleration of absorption, suggesting that parenteral treatment is still needed to cover the critical period following PPCI.

## Conclusions

An IA bolus of enoxaparin (0.75 mg/kg) followed by IV enoxaparin infusion (0.75 mg/kg/6 hours), in STEMI patients undergoing PPCI, appears to be suitable for further study to assess its efficacy and safety.
